# Tissue Factor Pathway Inhibitor 2 Is Found in Skin and Its C-Terminal Region Encodes for Antibacterial Activity

**DOI:** 10.1371/journal.pone.0052772

**Published:** 2012-12-26

**Authors:** Praveen Papareddy, Martina Kalle, Ole E. Sørensen, Katarina Lundqvist, Matthias Mörgelin, Martin Malmsten, Artur Schmidtchen

**Affiliations:** 1 Division of Dermatology and Venereology, Department of Clinical Sciences, Lund University, Biomedical Center, Lund, Sweden; 2 Division of Infection Medicine, Department of Clinical Sciences, Lund University, Biomedical Center, Lund, Sweden; 3 Department of Pharmacy, Uppsala University, Uppsala, Sweden; Institut de Pharmacologie et de Biologie Structurale, France

## Abstract

**Background:**

Tissue factor pathway inhibitor 2 (TFPI-2) is a matrix-associated serine protease inhibitor with an enigmatic function *in vivo*. Here, we describe that TFPI-2 is present in fibrin of wounds and also expressed in skin, where it is up-regulated upon wounding.

**Methodology and Principal Findings:**

Neutrophil elastase cleaved TFPI-2, and a C-terminal fragment was found to bind to bacteria. Similarly, a prototypic peptide representing this C-terminal part, EDC34, bound to bacteria and bacterial lipopolysaccharide, and induced bacterial permeabilization. The peptide also induced leakage in artificial liposomes, and displayed a random coil conformation upon interactions with liposomes as well as lipopolysaccharide. EDC34 was antibacterial against both Gram-negative and Gram-positive bacteria in physiological buffer conditions.

**Conclusions/Significance:**

The results demonstrate that the C-terminus of TFPI-2 encodes for antimicrobial activity, and may be released during wounding.

## Introduction

Antimicrobial peptides (AMP) provide a first line of defense against invading microbes, at epithelial surfaces as well as in blood [1], [2], [3]. Many antimicrobial peptides (AMPs), such as defensins and cathelicidins, are multifunctional and may exert immunomodulatory functions, and are therefore often denoted host defense peptides (HDP). HDPs have recently been shown to encompass not only the “classical” antimicrobial peptides, but also various bioactive peptides and proteins with antimicrobial activities, e.g., chemokines [4], ribonucleases [5], calgranulins [6], and complement C3a [7]. Furthermore, several proteins of the coagulation system, such as thrombin [8], kininogen [9], protein C inhibitor [10], fibrinogen [11], [12], and TFPI-1 [13], may either as intact molecules or after proteolysis, act in host defense involving killing of bacteria and immunomodulation. Tissue factor pathway inhibitor 2 (TFPI-2) is a 32 kDa matrix-associated Kunitz-type serine protease inhibitor. The molecule contains a highly negatively charged N-terminus, three tandemly-linked Kunitz-type domains, and a highly positively charged C-terminus [14], [15]. TFPI-2 is synthesized and secreted by many cells, including skin fibroblasts, endothelial cells, smooth muscle cells, dermal fibroblasts, keratinocytes, monocytes, and macrophages [16], [17], [18], [19], [20], [21]. *In vitro,* TFPI-2 weakly inhibits coagulation induced by the tissue factor (TF)-VII complex, and also inhibits a wide range of proteases like trypsin, chymotrypsin, plasmin, matrix metalloproteinases, factor XIa and plasma kallikrein [22], [23]. Notably, stimulation of human umbilical vein endothelial cells with inflammatory mediators such as phorbol myristate acetate, lipopolysaccharide (LPS), and TNF-α significantly increases TFPI-2 expression [17]. Analogously, in a murine model, TFPI-2 expression is dramatically upregulated in the liver during LPS stimulation [24]. Of note is that ADAMTS1 (A disintegrin and metalloproteinase with thrombospondin motifs 1), plasmin and thrombin have been shown to cleave TFPI-2 at its C-terminus *in vitro* [25].

The involvement of TFPI-2 in inflammation, as well as the presence of a highly cationic, protease releasable region, a feature typical for many host defense peptides, prompted us to investigate whether TFPI-2 is expressed in inflammatory settings, and whether its C-terminus may exhibit antimicrobial activities.

## Materials and Methods

### Ethics Statement

The procedures and use of human material was approved by the Ethics Committee at Lund University (LU 762-02, LU 509-01, LU 708-01).

### Peptides

The peptide EDC34 (EDCKRACAKALKKKKKMPKLRFASRIRKIRKKQF) was synthesized by Biopeptide Co., San Diego, CA, whereas LL-37 (LLGDFFRKSKEKIGKEFKRIVQRIKDFLRNLVPRTES), was obtained from Innovagen AB, Lund, Sweden. The purity (>95%) of these peptides was confirmed by mass spectral analysis (MALDI-ToF Voyager).

### Microorganisms

The bacterial isolates *E. coli* ATCC 25922, *P. aeruginosa* ATCC 27853, *P. aeruginosa* PA01, *Staphylococcus aureus* ATCC 29213, *Bacillus subtilis* ATCC 6633, *Candida albicans* ATCC 90028, and *Candida parapsilosis* ATCC 90018 were obtained from the American Type Culture Collection.

### Radial Diffusion Assay

Essentially as described earlier, bacteria were grown to mid-logarithmic phase in 10 ml of full-strength (3% w/v) trypticase soy broth (TSB) (Becton-Dickinson, Cockeysville, MD). The microorganisms were then washed once with 10 mM Tris, pH 7.4. Subsequently, 4×10^6^ bacterial colony forming units (cfu) were added to 15 ml of the underlay agarose gel, consisting of 0.03% (w/v) TSB, 1% (w/v) low electroendosmosis type (EEO) agarose (Sigma, St Louis MO) and 0.02% (v/v) Tween 20 (Sigma) with or without 0.15 M NaCl. The underlay was poured into a Ø 144 mm petri dish. After agarose solidification, 4 mm-diameter wells were punched and 6 µl of test sample was added to each well. Plates were incubated at 37°C for 3 hours to allow diffusion of the peptides. The underlay gel was then covered with 15 ml of molten overlay (6% TSB and 1% Low-EEO agarose in distilled H_2_O). Antimicrobial activity of a peptide is visualized as a zone of clearing around each well after 18–24 hours of incubation at 37°C.

### Viable Count Analysis (VCA)

*E. coli* and *S. aureus* strains were grown to mid-exponential phase in Todd-Hewitt (TH). *P. aeruginosa* and *C. albicans* strains were grown in Todd Hewitt broth (TH) overnight. Bacteria were washed and diluted in 10 mM Tris, pH 7.4, either alone or with 10 mM Tris, pH 7.4, containing 0.15 M NaCl. 2×10^6^ cfu/ml bacteria were incubated in 50 µl, at 37°C for 2 h with the C-terminal TFPI-2 derived peptide EDC34 at the indicated concentrations. Serial dilutions of the incubation mixture were plated on TH agar, followed by incubation at 37°C overnight and cfu determination.

### Slot-blot Assay

LPS binding of EDC34 and LL-37 was examined by a slot-blot assay. Peptides (1, 2 and 5 µg) were bound to nitrocellulose membrane (Hybond-C, GE Healthcare BioSciences Buckinghamshire, UK), pre-soaked in PBS, by vacuum. Membranes were then blocked by 2 wt% BSA in PBS, pH 7.4, for 1 h at RT and subsequently incubated with radiolabeled LPS (40 µg/mL; 0.13×10^6^ cpm/µg) for 1 h at RT in PBS. The radioiodination (^125^I) of LPS was performed as described earlier [8]. After LPS binding, the membranes were washed 3 times, 10 min each time, in PBS and visualized for radioactivity on Bas 2000 radio imaging system (Fuji, Tokyo, Japan). Measurements were performed also in the presence of unlabeled heparin (6 mg/ml) to test for competitive inhibition of peptide binding to LPS.

### Flow Cytometry Analysis

Fifty µl of bacteria (1–2×10^9 ^cfu) were incubated with 450 µl of human plasma or 10 mM Tris, pH 7.4, containing 0.15 M NaCl, either alone or supplemented with TAMRA-labeled EDC34 (at 3 µM). Samples were incubated for 30 min or 1 h at 37°C, then pelleted and washed twice with PBS before flow cytometry analysis was performed. Flow cytometry analysis (Becton-Dickinson, Franklin Lakes, NJ) was performed using a FACSCalibur flow cytometry system equipped with a 15 mW argon laser set a 488 nm. The bacterial population was selected by gating with appropriate settings of forward scatter (FSC) and sideward scatter (SSC). Controls without primary antibodies were included.

### Electron Microscopy

For transmission electron microscopy and visualization of peptide effects on bacteria, *P. aeruginosa* ATCC 27853 and *S. aureus* ATCC 29213 (1–2×10^6^ cfu/sample) were incubated for 2 h at 37°C with the peptides (30 µM). Samples of *P. aeruginosa* and *S. aureus* suspensions were adsorbed onto carbon-coated copper grids for 2 min, washed briefly by two drops of water, and negatively stained by two drops of 0.75% uranyl formate. The grids were rendered hydrophilic by glow discharge at low pressure in air. In another experiment, fibrin slough from patients with chronic venous ulcers (CWS) was fixed (1.5% PFA, 0.5% GA in 0.1 M phosphate buffer, pH 7.4) for 1 hour at room temperature, followed by washing with 0.1 M phosphate buffer, pH 7.4. The fixed and washed samples were subsequently dehydrated in ethanol and further processed for Lowicryl embedding [26]. Sections were cut with a LKB ultratome and mounted on gold grids. For immunostaining, the grids were floated on top of drops of immune reagents displayed on a sheet of parafilm. Free aldehyde groups were blocked with 50 mM glycine, and the grids were then incubated with 5% (vol/vol) goat serum in incubation buffer (0.2% BSA-c in PBS, pH 7.6) for 15 minutes. This blocking procedure was followed by overnight incubation at 4°C with 1 µg/ml of polyclonal rabbit antibodies raised against the C-terminal TFPI-2 sequence CAKALKKKKKMPKLRFASRIRKIRKKQF (CAK28 antibodies) (Innovagen AB). Controls without these primary antibodies were included. After washing the grids in a large volume (200 ml) of incubation buffer, floating on drops containing the gold conjugate reagents, 1 µg/ml EM goat antiRabbit IgG 10 nm Au (BBI) in incubation buffer was performed for 2 h at 4°C. After further washes by an excess volume of incubation buffer, the sections were postfixed in 2% glutaraldehyde. Finally, sections were washed with distilled water and poststained with 2% uranyl acetate and lead citrate. All samples were examined with a Jeol JEM 1230 electron microscope operated at 80 kV accelerating voltage. Images were recorded with a Gatan Multiscan 791 charge-coupled device camera.

### Histochemistry

For immunostaining, wound biopsies were fixed in 10% formalin, rehydrated and embedded in paraffin. Sections of 5 µm thickness were placed on poly-lysine coated glass slides, deparaffinized in xylene and rehydrated in graded alcohols. The slides were then treated with Dako antigen retrieval solution (Dako) for 40 min at 97°C, and incubated for 24 h at room temperature in a 1∶50 dilution of C-terminal polyclonal antibodies against TFPI-2 (CAK28 antibodies) diluted in TBS with 1% BSA, 5% goat serum, and 0.05% Tween 20. After three 20 min washes in TBS with 0.05% Tween 20, the sections were incubated with alkaline phosphatase conjugated secondary goat anti-rabbit IgG (Dako) diluted 1∶1000 in the same buffer as the primary antibody and incubated for another 24 hours, followed by three 20 min washes. Sections were developed with Vulcan Fast Red chromogen (Biocare Medical, Concord, CA) and the slides were counterstained with Harris Hematoxylin (EM Science, Gibbstown, NJ). Excess EDC34 peptide added with the TFPI-2 antibodies blocked the staining (Figure S1).

### Human Skin Wounds

Non-wounded human skin was obtained by taking punch biopsies from three donors, while skin wound samples were retrieved by making new punch biopsies from the edges of the initial biopsies. A small fraction of these samples were fixed in formalin for immunohistochemistry or prepared for microarray analysis as previously described [27]. The material was obtained under protocols approved by the Ethics Committee at Lund University, Lund, Sweden.

### Model of *ex vivo* Injured Human Skin

Surplus, normal, skin was obtained as previously described from three donors following surgery according to protocols approved by the Ethics Committee at Lund University. In brief the skin was cut into slices and incubated in culture for 4 days (*ex vivo* injured skin). The skin samples were cultured in serum-free keratinocyte medium (KGM2-Bullet kit) from Cambrex (Walkersville, MD) supplemented with transferrin, hEGF (0.15 ng/mL), 0.5 mg/mL hydrocortisone, gentamicin, amphotericin B, and epinephrine but without insulin (all supplied by Cambrex).

### RNA Isolation and Microarray Analysis

Total RNA was isolated with Trizol (Invitrogen, Carlsbad, CA) according to the recommendations of the supplier and resuspended in 0.1 mmol/L EDTA. The concentration was determined by spectrophotometric measurement. For gene expression analysis, total RNA was biotinylated and hybridized to Human Genome U133 Plus 2.0 GeneChips*H* (Affymetrix, Santa Clara, CA) according to the instructions by the manufacturer. The microarray fluorescence signals were normalized using the GeneChip Operation Software (GCOS ver. 1.4, Affymetrix). All probe set lists were annotated with locus link identifications (IDs) provided by the NetAffx database and converged into gene/EST (expressed sequence tag) lists by exclusion of redundant probe sets with identical locus ID. Genes were defined as expressed in cell populations if all replicates were assigned a present call by the GCOS software (Affymetrix). Genes of potential interest for wound healing were therefore only genes with at least three present calls in either the *in vivo* control condition or in the *in vivo* wound condition. The microarray data present in this study has been sent to the MAIME (Minimum Information About a Microarray Experiment) compliant MAGE-TAB format to the ArrayExpress database (www.ebi.ac.uk/arrayexpress) and are publicly available under the accession number E-MEXP-3305 [28].

### Protein Digestion

recTFPI-2 (GenWay Biotech, Inc.) was digested with human leukocyte elastase (Calbiochem®) (1∶1) for 4 and 8 h at 37°C. Digested samples were analyzed by SDS-PAGE and western blot. Proteins and peptides were transferred to nitrocellulose membranes (Hybond-C). Membranes were blocked by 3% (w/v) skimmed milk, washed, and incubated for 1 h with rabbit anti-human C-terminal TFPI-2 polyclonal antibodies (CAK28 antibodies, 1∶1000), washed three times for 10 min, and subsequently incubated (1 h) with HRP-conjugated secondary antibodies (1∶2000) (Dako), and then washed again three times, each time for 10 min. TFPI-2 and related fragments were visualized using the SuperSignal West Pico Chemiluminescent Substrate developing system (Thermo scientific). For N-terminal sequencing, digested samples were transferred to a PVDF-P^sq^ (Millipore) membrane and were analyzed at Proteomics Karolinska Institutet (PK/PI). In another experiment, digested and undigested samples were incubated with 50 µl of *E. coli* (1×10^9^ cfu/ml) for 1 h. Unbound supernatants (S) and bacterial bound protein pellet (P) samples was prepared and analyzed as above.

### Hemolysis Assay

EDTA-blood was centrifuged at 800 g for 10 min, where after plasma and buffy coat were removed. The erythrocytes were washed three times and resuspended in PBS, pH 7.4 to get a 5% suspension. The cells were then incubated with end-over-end rotation for 60 min at 37°C in the presence of peptides at the indicated concentrations. 2% Triton X-100 (Sigma-Aldrich) served as positive control. The samples were then centrifuged at 800 g for 10 min and the supernatant was transferred to a 96 well microtiter plate. The absorbance of hemoglobin release was measured at 540 nm and expressed as % of Triton X-100 induced hemolysis.

### Liposome Preparation and Leakage Assay

The liposomes investigated were anionic (DOPE/DOPG 75/25 mol/mol). DOPG (1,2-Dioleoyl-*sn*-Glycero-3-Phosphoglycerol, monosodium salt) and DOPE (1,2-dioleoyl-*sn*-Glycero-3-phoshoetanolamine) were both from Avanti Polar Lipids (Alabaster, USA) and of >99% purity. Due to the long, symmetric and unsaturated acyl chains of these phospholipids, several methodological advantages are reached. In particular, membrane cohesion is good, which facilitates very stable, unilamellar, and largely defect-free liposomes (observed from cryo-TEM), allowing detailed studies on liposome leakage. The lipid mixtures were dissolved in chloroform, after which solvent was removed by evaporation under vacuum overnight. Subsequently, 10 mM Tris buffer, pH 7.4, was added together with 0.1 M carboxyfluorescein (CF) (Sigma, St. Louis, USA). After hydration, the lipid mixture was subjected to eight freeze-thaw cycles consisting of freezing in liquid nitrogen and heating to 60°C. Unilamellar liposomes of about Ø140 nm were generated by multiple extrusions through polycarbonate filters (pore size 100 nm) mounted in a LipoFast miniextruder (Avestin, Ottawa, Canada) at 22°C. Untrapped CF was removed by two subsequent gel filtrations (Sephadex G-50, GE Healthcare, Uppsala, Sweden) at 22°C, with Tris buffer as eluent. CF release from the liposomes was determined by monitoring the emitted fluorescence at 520 nm from a liposome dispersion (10 µM lipid in 10 mM Tris, pH 7.4). An absolute leakage scale was obtained by disrupting the liposomes at the end of each experiment through addition of 0.8 mM Triton X-100 (Sigma-Aldrich, St. Louis, USA). A SPEX-fluorolog 1650 0.22-m double spectrometer (SPEX Industries, Edison, USA) was used for the liposome leakage assay. Measurements were performed in triplicate at 37°C.

### CD-spectroscopy

Circular dichroism (CD) spectra were measured by a Jasco J-810 Spectropolarimeter (Jasco, Easton, USA). The measurements were performed in duplicate at 37°C in a 10 mm quartz cuvette under stirring with a peptide concentration of 10 µM. The effect on peptide secondary structure of liposomes at a lipid concentration of 100 µM was monitored in the range 200–260 nm. To account for instrumental differences between measurements, as well as signals from bulk solution, background subtraction was performed routinely.

## Results

### Generation of C-terminal TFPI-fragments *in vitro* and *in vivo*

**Figure 1:**
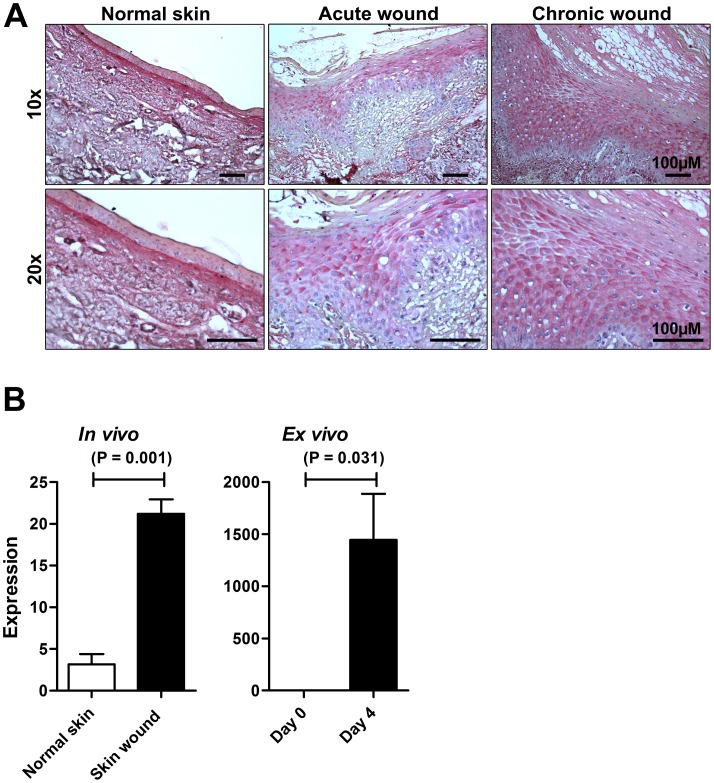
Identification and expression of TFPI-2 in human skin and wounds. (**A**) Immunohistochemical identification of TFPI-2 in normal skin, acute skin wound and in chronic venous leg ulcer tissue (chronic wound). Skin biopsies were taken from normal skin (n = 3), acute wounds (n = 3) and from the wound edges of patients with chronic venous ulcers (n = 3). Representative sections are shown. Scale bar is 100 µm. (**B**) TFPI-2 expression levels in wounds *in vivo* and *ex vivo*. Expression levels of TFPI-2, as determined by array data, in wounded and non-wounded skin are presented (n = 3).

**Figure 2:**
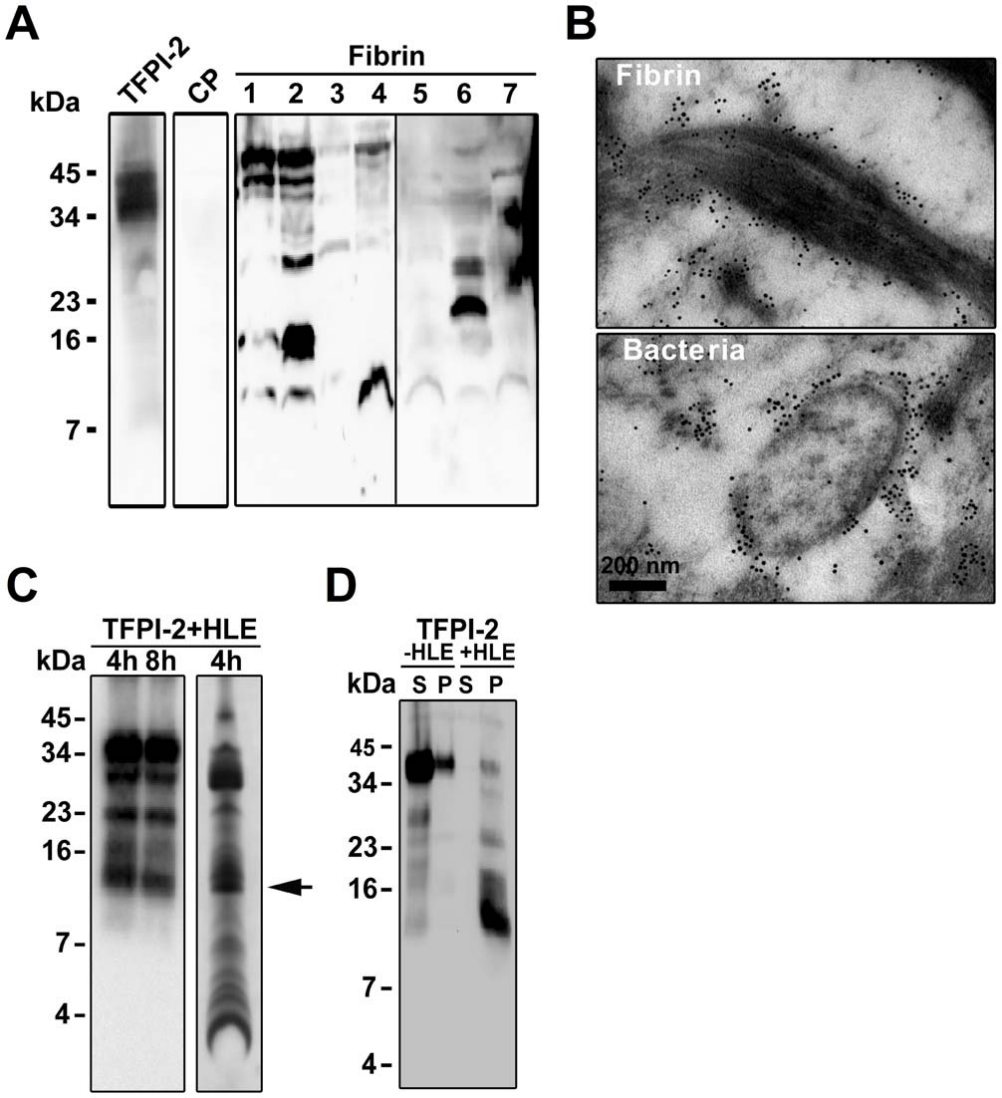
Identification of TFPI-2 cleavage fragments in wounds. (**A**) Fibrin clots obtained from patients with chronic wounds were analyzed by immunoblotting using polyclonal antibodies against the C-terminal of TFPI-2. Citrate plasma (CP) and recTFPI-2 were used as negative and positive controls. (**B**) TFPI-2 C-terminal epitopes are found in human wounds. Gold-labeled antibodies against the C-terminal of TFPI-2 (visualized as black dots) show that this epitope is found on bacteria present in fibrin slough from a *P. aeruginosa* infected chronic wound. (**C**) Proteolytic cleavage of recTFPI-2 with human leukocyte elastase (HLE). Left panel: Western blot analysis of degraded protein using polyclonal antibodies against the C-terminal part of TFPI-2. Right panel: Coomassie stained gel of the same material. The arrow indicates the sequenced fragment. (**D**) Digested C-terminal TFPI-2 fragment binds to bacterial surface. Intact and cleaved recTFPI-2 was incubated with *E. coli* for 1 h at 37°C and supernatants (S) and bacterial bound proteins were (pellet; P) analyzed by western blot.

As mentioned above, TFPI-2 is produced by many mesenchymal end epithelial cell types [18], [19], [20], [21]. In line with these findings, immunohistochemistry analyses showed that TFPI-2 was present in normal human skin, and was particularly found in the upper dermal layers, while the staining was found to be lower in the epidermis, particularly in the basal and suprabasal regions **(** **Fig. 1A** **, see also Fig. S1 and Table S1)**. TFPI-2 was furthermore detected in human skin wounds. Notably, it was observed in association with wound edges, and was noted also in the inflammatory infiltrate **(** **Fig. 1A** **, Fig. S1 and Table S1)**. TFPI-2 mRNA levels were significantly increased during wounding of skin, both *in vivo* as well as *ex vivo* **(** **Fig. 1B** **)**. Immunoblotting using antibodies against the C-terminal part of TFPI-2 identified the intact molecule along with C-terminal fragments of 7–16 kDa (and higher) in fibrin slough from a chronic wound of venous origin **(** **Fig. 2A** **)**. Electron microscopy analysis of this fibrin slough, using gold-labeled antibodies, showed that C-terminal TFPI-2 peptide epitopes were particularly associated with bacteria and fibrin fibers in these wounds **(** **Fig. 2B** **)**. *In vitro*, digestion of TFPI-2 with human neutrophil elastase, was found to generate a major C-terminal TFPI-2 fragment of about 10–16 kDa (**Fig. 2C**, arrow), similar in size to those identified in fibrin *in vivo*. N-terminal sequencing of this fragment identified that the enzyme is able to cleave after position 184 in the intact TFPI-2 molecule. Furthermore, this fragment was found to bind to bacteria in a pull-down assay **(** **Fig. 2D** **)**. Taken together, these results demonstrate that TFPI-2 is present in skin and wounds, and that C-terminal epitopes of the molecule are detected *in vivo*, particularly in association with fibrin but also on bacterial surfaces. Correspondingly, C-terminal fragments of pure TFPI-2 were generated *in vitro* by neutrophil elastase, and were found to bind to bacteria.

### Membrane Activities of a Prototypic C-terminal TFPI-2 Derived Peptide, EDC34

**Figure 3:**
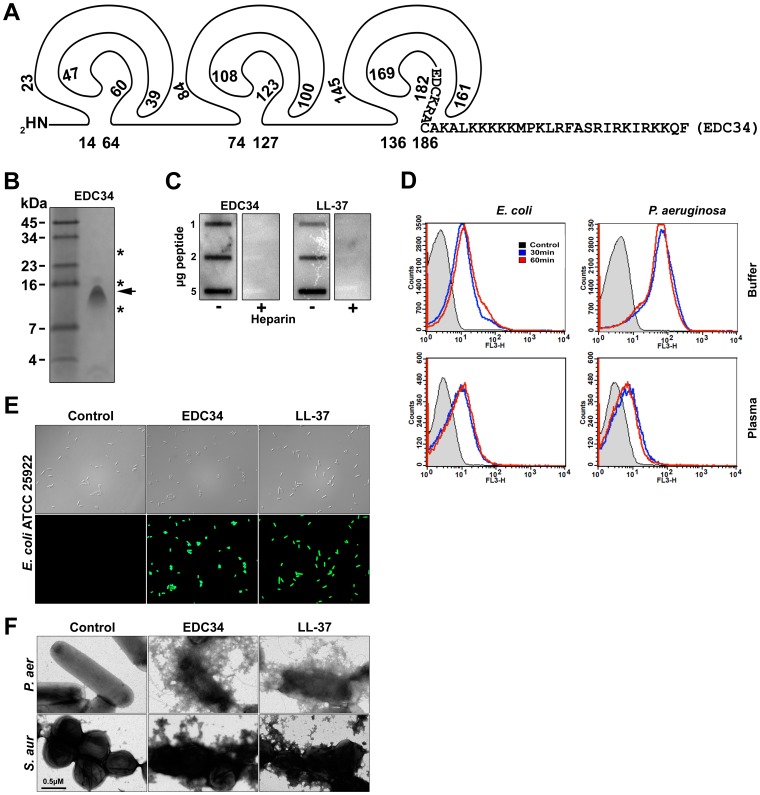
Activities of the C-terminal TFPI-2 peptide (EDC34). (**A**) Schematic representation of TFPI-2. The EDC34 peptide sequence is indicated. (**B**) Migration of EDC34 on 16.5% SDS-PAGE. The arrow indicates the position of the neutrophil elastase generated C-terminal peptide, whereas the asterisks indicate the positions of C-terminal fragments found in human fibrin slough. (**C**) LPS-binding activity of EDC34. Peptides were applied to a nitrocellulose membrane followed by incubation in PBS (containing 2% bovine serum albumin) with iodinated (^125^I)-LPS. Similar to LL-37, the EDC34 peptide bound LPS. The binding was inhibited by heparin (6 mg/ml). (**D**) Interaction of EDC34 with bacterial surfaces. The indicated bacteria (1–2×10^9^ cfu/ml, 50 µl) were incubated with 3 µM TAMRA-labeled EDC34 in 450 µl of 10 mM Tris, pH 7.4, 0.15 M NaCl (Buffer), or 450 µl of human citrate-plasma (Plasma), and samples were analyzed by FACS. (**E**) Permeabilizing effects of peptides on *E. coli*. Bacteria were incubated with 30 µM of EDC34 or LL-37, and permeabilization was assessed using the impermeant probe FITC. (**F**) Electron microscopy analysis. *P. aeruginosa* and *S. aureus* bacteria were incubated for 2 h at 37°C with 30 µM of EDC34 and LL-37 and visualized by negative staining. Control; buffer control.

To elucidate potential antimicrobial effect of the C-terminal region of TFPI-2, we synthesized the representative peptide EDC34, spanning the C-terminal cationic part of the molecule **(**for sequence see **Fig. 3A** **)**, comprising the peptide sequence region released by neutrophil elastase *in vitro*, and migrating on SDS-PAGE similarly to i) the low molecular weight C-terminal TFPI-2 fragments observed in fibrin slough from human wounds, and ii) the major C-terminal fragment detected after digestion of TFPI-2 with neutrophil elastase **(**compare **Fig. 2C, D** and **Fig. 3B** **)**. Initial experiments using a slot-binding assay demonstrated that the peptide avidly bound to LPS from *E. coli*, in a similar way as observed for the classical antimicrobial peptide LL-37. This binding was completely blocked by heparin **(** **Fig. 3C** **)**. Further experiments using FACS showed that TAMRA-labeled EDC34 was capable of binding to both *E. coli* and *P. aeruginosa* in physiological salt buffer as well as in presence of human plasma **(** **Fig. 3D** **)**, results compatible with the above observed binding of the neutrophil elastase generated C-terminal TFPI-2 fragment to *E. coli* **(** **Fig. 2D** **)**. Correspondingly, studies employing the impermeant probe FITC showed that EDC34 permeabilized *E. coli* membranes similarly to LL-37 [2], [29] **(** **Fig. 3E** **)**. Electron microscopy utilizing *P. aeruginosa* and *S. aureus* demonstrated that EDC34 caused extensive membrane damage, characterized by membrane breaks and leakage of intracellular material into the extracellular compartment **(** **Fig. 3F** **)**.

**Figure 4:**
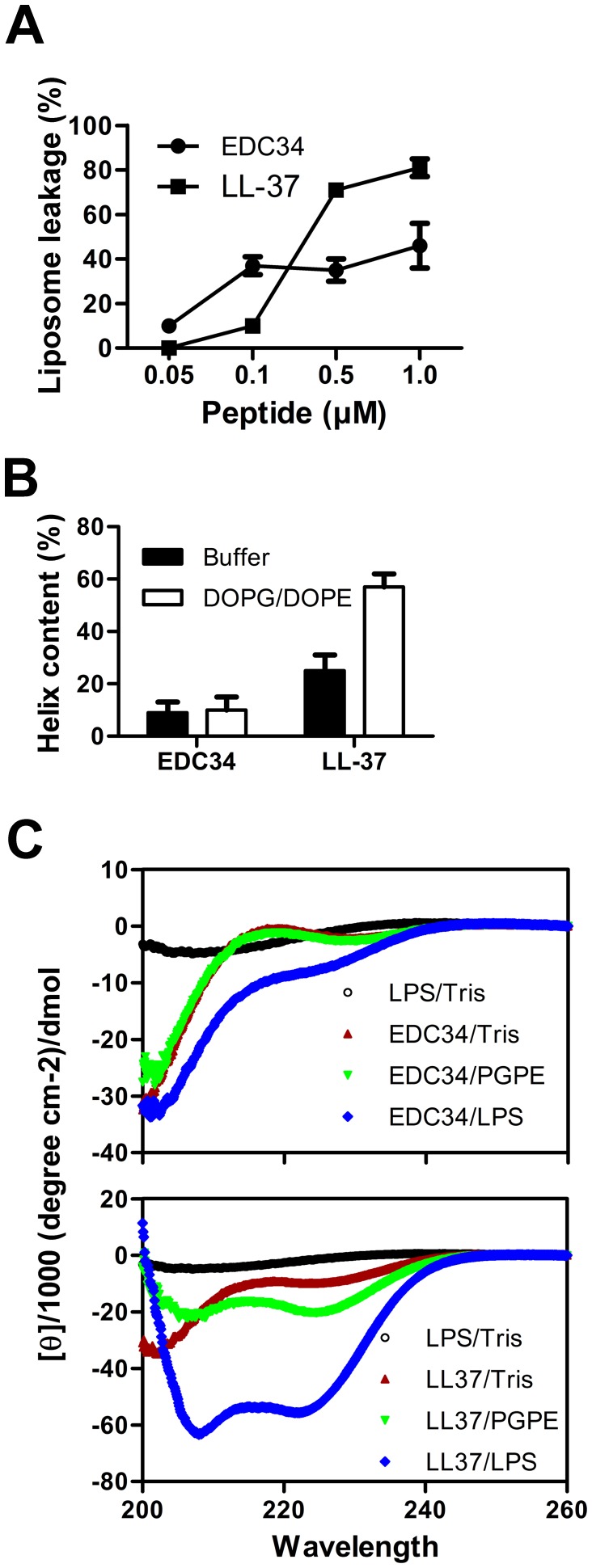
Peptide effects on liposomes and biophysical studies. (**A**) The membrane permeabilizing effect of EDC34 and LL-37 was recorded by measuring fluorescence release of carboxyfluorescein from PA (negatively charged) liposomes. The experiments were performed in 10 mM Tris buffer. Values represents mean of triplicate samples. (**B**) Helical content of EDC34 and LL-37 peptides in presence of negatively charged liposomes (PA). The structure of EDC34 was largely unaffected by the addition of liposomes. (**C**) CD spectra of EDC34 and LL-37 in Tris buffer and in presence of LPS. For control, CD spectra for buffer and LPS alone are also presented.

**Figure 5:**
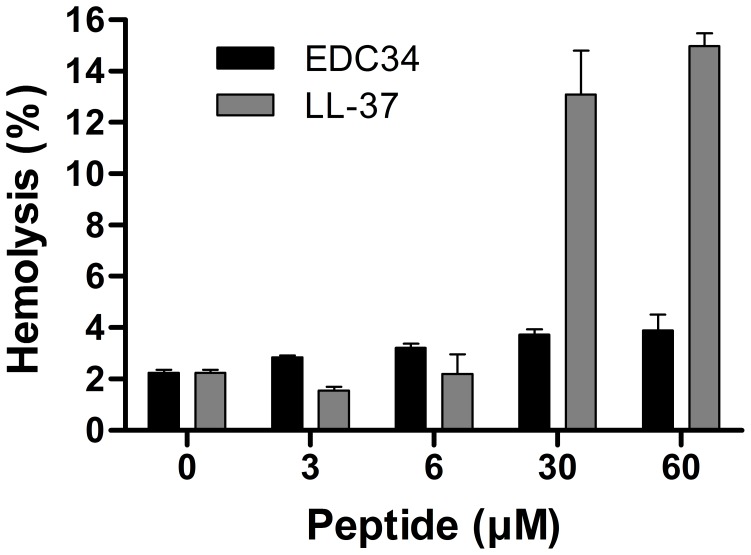
Activities on eukaryotic cells. Hemolytic effects of the indicated peptides. The cells were incubated with different concentrations of the peptides, 2% Triton X-100 (Sigma-Aldrich) served as positive control. The absorbance of hemoglobin release was measured at λ 540 nm and is expressed as % of Triton X-100 induced hemolysis (note the scale of the y-axis). Effects of LL-37 are shown for comparison.

Further mechanistic studies using a liposome model were employed in order to study membrane permeabilisation by EDC34. The peptide caused carboxyfluorescein (CF) release from DOPE/DOPG liposomes, indicating an effect on lipid membranes, although to a substantially lesser extent than observed for LL-37 **(** **Fig. 4A** **)**. No significant conformational changes were found on binding to liposomes **(** **Fig. 4B** **)** or *E. coli* LPS **(** **Fig. 4C** **)**. This in contrast to LL-37, which showed a significant increase in helicity in both cases. Finally, studies showed that the peptide did not display significant hemolytic activity at doses of 3–60 µM **(** **Fig. 5** **)**. Taken together, these data above indicate that EDC34, although showing rather low permeabilizing activity on DOPG/DOPE liposomes and erythrocytes, may interact with both LPS and bacterial membranes, leading to bacterial permeabilization and disintegration of bacteria.

### Antimicrobial Effects of EDC34

**Figure 6:**
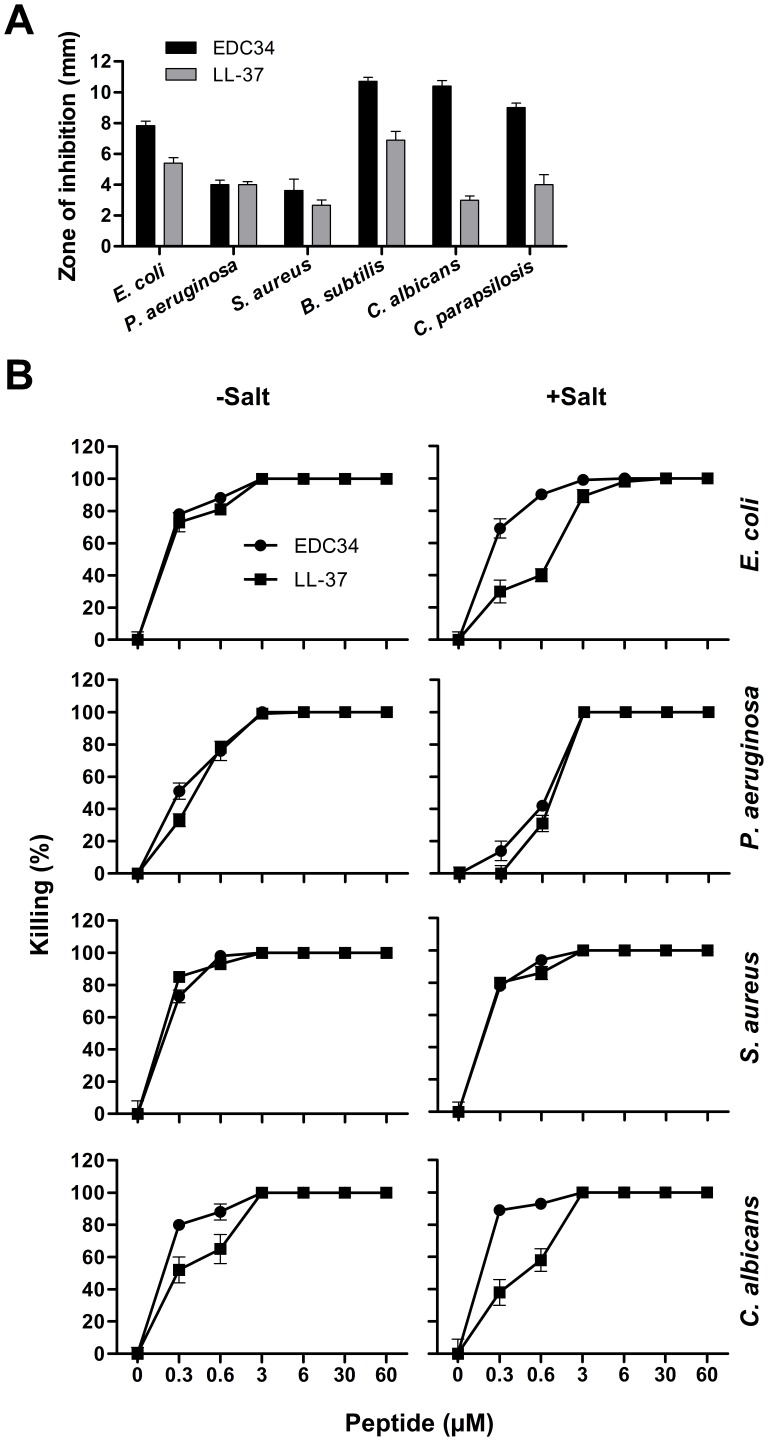
Antimicrobial activities of EDC34. (**A**) Antimicrobial activity of EDC34 (at 100 µM in RDA) was tested against the indicated microbes. For determination of effects, *E. coli* ATCC 25922, *P. aeruginosa* ATCC 27853, *S. aureus* ATCC 29213 or *B. subtilis* ATCC 6633 isolates (4×10^6^ cfu) or C. *albicans* ATCC 90028 and *C. parapsilosis* ATCC 90018 (1×10^5^ cfu) were inoculated in 0.1% TSB agarose gel. Each 4 mm-diameter well was loaded with 6 µl of peptide. The zones of clearance correspond to the inhibitory effect of each peptide after incubation at 37°C for 18–24 h. LL-37 (at 100 µM) was used for control (mean values are presented, n = 3). (**B**) Antibacterial effects of EDC34 and LL-37 against the indicated bacterial strains in viable count assays. 2×10^6^ cfu/ml bacteria were incubated in 50 µl with peptides at the indicated concentrations in 10 m.

Studies using RDA and the Gram-negative *E. coli* and *P. aeruginosa,* Gram-positive *Bacillus subtilis* and *Staphylococcus aureus* as well as the fungi *Candida albicans* and *Candida parapsilosis*, demonstrated inhibitory effects of EDC34 on these microbes **(** **Fig. 6A** **)**. These studies were further substantiated by matrix-free viable count assays. Experiments utilizing *E. coli*, *P. aeruginosa, S. aureus* and *C. albicans* strains, respectively, demonstrated that when compared to LL-37, EDC34 displayed a significant antibacterial activity at doses of 0.3–0.6 µM, which was retained in presence of physiological salt conditions **(** **Fig. 6B** **)**. Notably, intact recTFPI-2 did not show any antimicrobial effects in RDA and viable count assays (data not shown).

## Discussion

TFPI-2 is found in a variety of tissues, particularly in the extracellular matrix. The identification of TFPI-2 in skin, as well as in wounds, is therefore well in line with previous observations, and extend these into a setting of skin wounding and bacterial infection. Although our data correspond to a report by Torres-Collado [25], indicating possible proteolysis of TFPI-2, it is of note that C-terminal fragments have not been previously described. Therefore, our findings on the generation of these epitopes, both *in vivo* in fibrin, and *in vitro* after digestion with neutrophil elastase, are particularly relevant in the context of proteolysis of TFPI-2 and possible release of bioactive host defense fragments. Notably, neutrophil elastase was found to degrade TFPI-2 *in vitro*, generating a C-terminal fragment comparable in size to those found *in vivo*. However, the corresponding sequences of C-terminal TFPI-2 peptides detected *in vivo*, as well as their possible generation by proteolysis of TFPI-2 by thrombin, plasmin, as well as other enzymes clearly remain to be defined.

From a structural point of view, EDC34 is related to a similar cationic region in TFPI-1. This protein, which is to a large extent present in vascular tissue, but also skin [13], also releases C-terminal fragments, constituting for example a 27-mer antimicrobial peptide. **Table 1** illustrates that the two peptides, although demonstrating a low homology, share a similar overall overrepresentation of K and R residues. Interestingly, the TFPI-2-derived peptide EDC34 is of higher charge, and contains two cationic stretches in contrast to the single stretch observed in the TFPI-1 peptide GGL27.

**Table 1:** C-terminal sequences of human TFPI-1 and -2.

Protein	Sequence	Charge
TFPI-1	**QECLRACKKGFIQRISKGGLIKTKRKRKKQRVKIAYEEIFVKNM**	+12
TFPI-2	**EDCKRACAKALKKKKKMPKLRFASRIRKIRKKQF–––---**	+14

In contrast to LL-37, EDC34 displayed a low helical content in buffer and in presence LPS, reflecting its low content of features typical of “classical” helical peptides, such as regularly interspersed hydrophobic residues. Evidently, the mechanism of action involves a direct bacterial killing, most likely by bacterial membrane disruption. A number of mechanisms by which AMPs induce membrane defects have been observed. For some peptides, such as LL-37, melittin, alamethicin, magainin 2 and gramicidin A [30], [31], [32], [33], transmembrane structures have been reported, which are often associated with an ordered secondary structure in the membrane-bound peptide. For disordered and highly charged peptides, membrane disruption is likely obtained by other mechanisms, e.g., induction of a negative curvature strain, membrane thinning, or local packing defects associated with peptide localization primarily in the phospholipid polar head group region [30], [34], [35], [36], [37], [38]. Interestingly, biophysical studies on a kininogen-derived antimicrobial peptide, HKH20 (HKHGHGHGKHKNKGKKNGKH) [39], showed that the HKH20 peptide, like EDC34, displays primarily random coil conformation in buffer and at lipid bilayers, having interactions with the membrane dominated by electrostatics [36]. Nevertheless, irrespective of the mode of action, it is notable that EDC34 showed a similar efficiency as the classical LL-37 in killing *P. aeruginosa* and *S. aureus* at physiological salt strength.

In summary, the present results demonstrate the presence of TFPI-2 in skin and wounds, as well as its up-regulation during wounding. The fact that neutrophil elastase generated a C-terminal TFPI-2 fragment which interacted with bacteria *in vitro*, and that similar fragments were found *in vivo* and in association with bacteria, indicated a plausible antimicrobial role of the C-terminal region of TFPI-2. A prototypic fragment of TFPI-2 was found to exert antimicrobial effects similar to LL-37 at physiological conditions. Further studies are needed in order to define the exact peptide sequences present *in vivo*. Furthermore, considering the multiple roles of host defense peptides, this particular region of TFPI-2 may also exert other bioactive roles. For example, recent data indicate that EDC34, like the related peptide of TFPI-1 [13], may in addition to its direct effects on bacteria, boost complement activation on Gram-negative bacteria (Papareddy et al., in manuscript). Furthermore, the present data do not disclose whether TFPI-2 confers protection to bacterial infection *in vivo*. Although of clear relevance, such studies employing TFPI-2 deficient mice are well beyond the scope of this current report.

## Supporting Information

Figure S1 **Controls to** **Figure 1** **.** Immunohistochemical identification of TFPI-2 in sections from an acute wound edge is shown in the left panel (TFPI-2). In Control 1, the staining was blocked by excess EDC34 peptide added with the primary antibodies. In Control 2, only secondary antibodies were used.
(TIF)

Table S1 **Skin biopsies were taken from normal skin (n = 3), acute wounds (n = 3) and from the wound edges of patients with chronic venous ulcers (n = 3).** The staining for TFPI-2 was evaluated using two magnifications (10 and 20x) by two observers. –; no staining, +; weak staining, ++; moderate staining, +++; strong staining. Scale bar is 100 µm. Since the staining was found to be localized, the terms “upper” and “lower” dermis fairly well relate to papillary and reticular dermis, and the same applies to epidermis, relating to basal and suprabasal layers and upper, superficial layers involving stratum granulosum, respectively.
(DOCX)
