# Supplementary figures and images for: Tissue Factor Pathway Inhibitor 2 Is Found in Skin and Its C-Terminal Region Encodes for Antibacterial Activity
Source: PLoS One. 2012 Dec 26;7(12):e52772. doi: 10.1371/journal.pone.0052772 (PMC3530512; doi:10.1371/journal.pone.0052772)

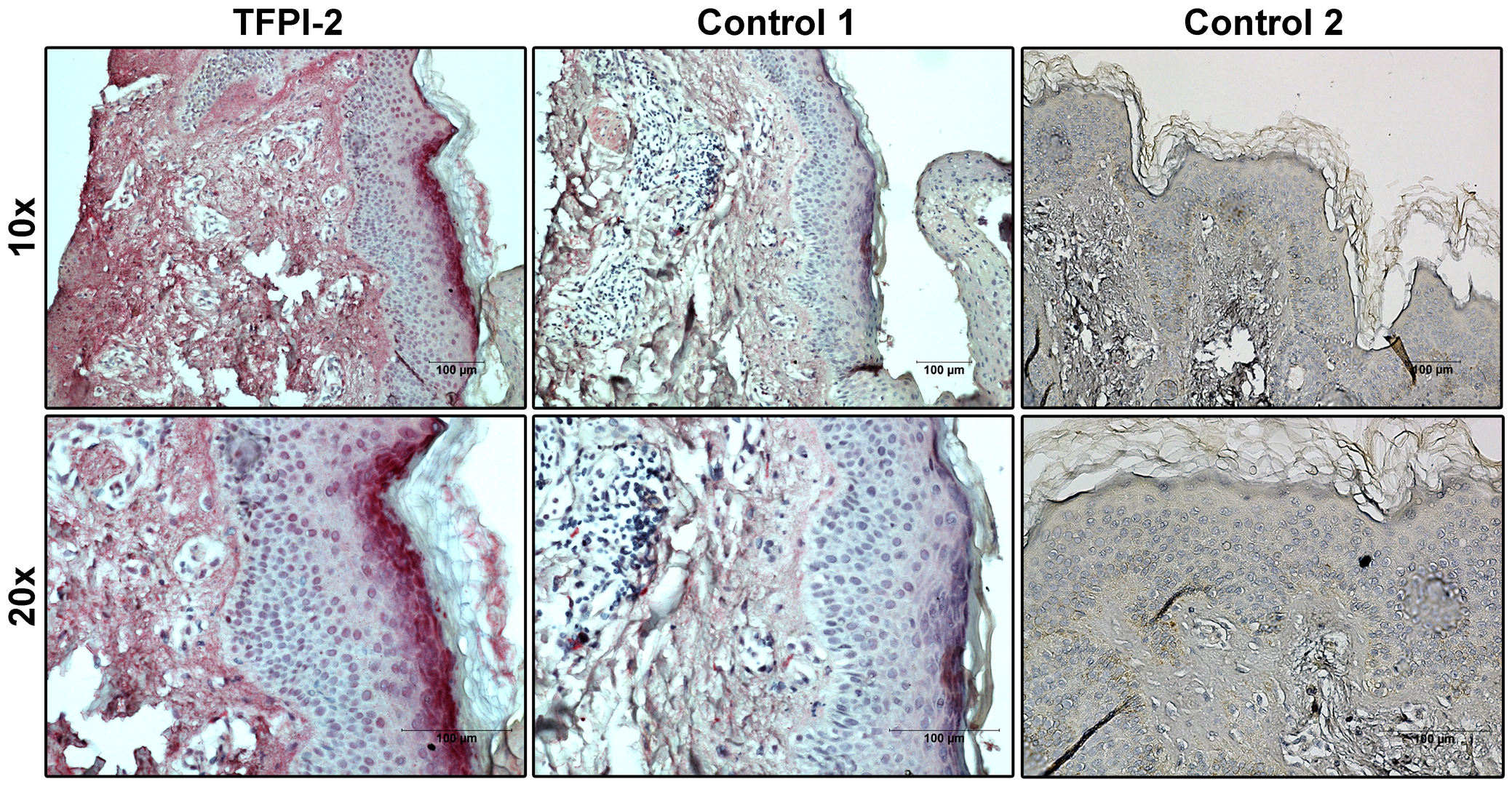

Supplement: Figure S1 — Controls to Figure 1 . Immunohistochemical identification of TFPI-2 in sections from an acute wound edge is shown in the left panel (TFPI-2). In Control 1, the staining was blocked by excess EDC34 peptide added with the primary antibodies. In Control 2, only secondary antibodies were used. (TIF) [file pone.0052772.s001.tif]
