# Supplementary material for: Tissue Factor Pathway Inhibitor 2 Is Found in Skin and Its C-Terminal Region Encodes for Antibacterial Activity
Source: PLoS One. 2012 Dec 26;7(12):e52772. doi: 10.1371/journal.pone.0052772 (PMC3530512; doi:10.1371/journal.pone.0052772)
Supplement: Table S1 — Skin biopsies were taken from normal skin (n = 3), acute wounds (n = 3) and from the wound edges of patients with chronic venous ulcers (n = 3). The staining for TFPI-2 was evaluated using two magnifications (10 and 20x) by two observers. –; no staining, +; weak staining, ++; moderate staining, +++; strong staining. Scale bar is 100 µm. Since the staining was found to be localized, the terms “upper” and “lower” dermis fairly well relate to papillary and reticular dermis, and the same applies to epidermis, relating to basal and suprabasal layers and upper, superficial layers involving stratum granulosum, respectively. (DOCX) [file pone.0052772.s002.docx]

| **Patient** | **Epidermis** | | | | | | **Dermis** | | | | | |
| --- | --- | --- | --- | --- | --- | --- | --- | --- | --- | --- | --- | --- |
|  | **Upper** | | | **Lower** | | | **Upper** | | | **Lower** | | |
|  | **1** | **2** | **3** | **1** | **2** | **3** | **1** | **2** | **3** | **1** | **2** | **3** |
| **Normal skin** | **+** | **+** | **+** | **-** | **-** | **-** | **++** | **++** | **+** | **+** | **-** | **-** |
| **Acute wound** | **++** | **++** | **++** | **-** | **-** | **-** | **++** | **++** | **++** | **+** | **+** | **+** |
| **Chronic wound** | **++** | **++** | **++** | **-** | **+** | **-** | **++** | **+++** | **++** | **+** | **++** | **+** |
